# A Structural Study on Absorption of Lysozyme in Amorphous Starch Microspheres

**DOI:** 10.1021/acs.molpharmaceut.4c00135

**Published:** 2024-05-13

**Authors:** Henrik Vinther So̷rensen, Nedim Krcic, Ian George, Vitaly Kocherbitov

**Affiliations:** † Department of Biomedical Science, Faculty of Health and Society, Malmö University, Malmö 20506, Sweden; ‡ Biofilms Research Center for Biointerfaces, Malmö University, Malmö 20506, Sweden; § Magle Chemoswed AB, Agneslundsvägen 27, Malmö 21215, Sweden

**Keywords:** small-angle X-ray scattering, lysozyme, starch microspheres, drug delivery

## Abstract

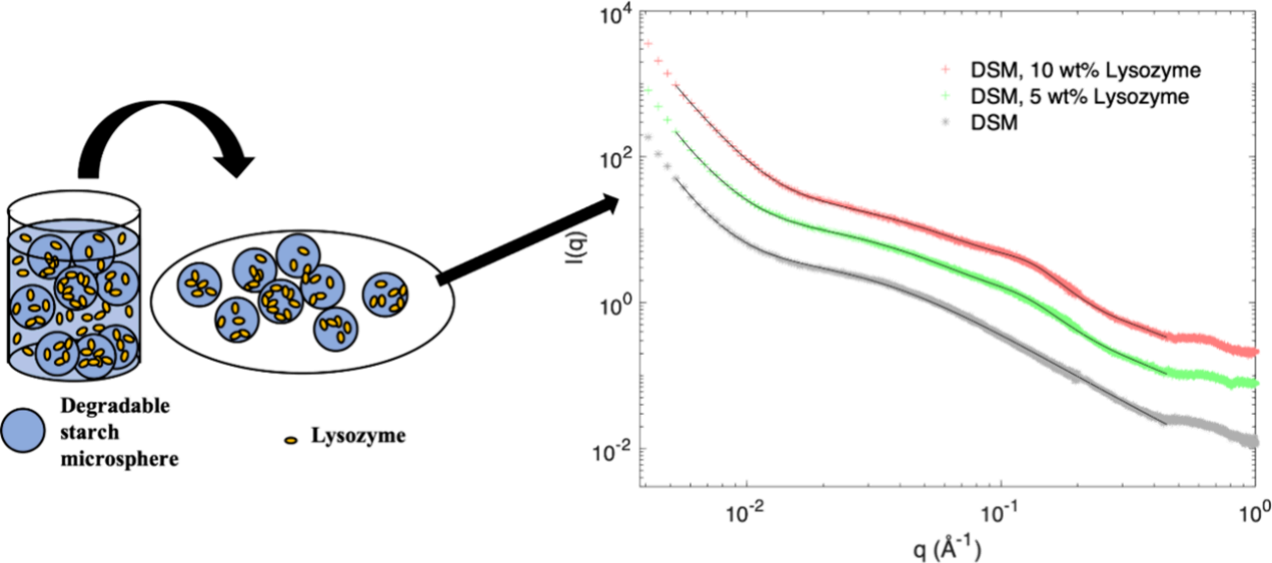

The potential of using proteins as drugs is held back by their low stability in the human body and challenge of delivering them to the site of function. Extensive research is focused on drug delivery systems that can protect, carry, and release proteins in a controlled manner. Of high potential are cross-linked degradable starch microspheres (DSMs), as production of these is low-cost and environmentally friendly, and the products are degradable by the human body. Here, we demonstrate that DSMs can absorb the model protein lysozyme from an aqueous solution. At low amounts of lysozyme, its concentration in starch microspheres strongly exceeds the bulk concentration in water. However, at higher protein contents, the difference between concentrations in the two phases becomes small. This indicates that, at lower lysozyme contents, the absorption is driven by protein–starch interactions, which are counteracted by protein–protein electrostatic repulsion at high concentrations. By applying small-angle X-ray scattering (SAXS) to the DSM–lysozyme system, we show that lysozyme molecules are largely unaltered by the absorption in DSM. In the same process, the starch network is slightly perturbed, as demonstrated by a decrease in the characteristic chain to chain distance. The SAXS data modeling suggests an uneven distribution of the protein within the DSM particles, which can be dependent on the internal DSM structure and on the physical interactions between the components. The results presented here show that lysozyme can be incorporated into degradable starch microspheres without any dependence on electrostatic or specific interactions, suggesting that similar absorption would be possible for pharmaceutical proteins.

## 1 Introduction

Since proteins exhibit very specific functions for their host organisms, they have high potential as pharmaceutical drugs, and they can be used in disease treatment with only limited side effects.^1,2^ Unfortunately, poor stability, high degradability, and difficulties in delivering the proteins to the site of action have limited the use of proteins for pharmaceutical purposes.^3^ This has sparked great interest in developing protein delivery systems that can encapsulate proteins and allow a controlled release. The optimal drug carrier is made of abundant, natural, environmentally friendly material that is cheap and biocompatible. A material that fits this description is starch, which is one of the most abundant biopolymers in the world. Starch is a common component of human foods, so it can easily be degraded by the digestive system. Smaller starch quantities can even be degraded in the blood, where amylase can convert it to smaller sugars.^4^

Starch is made of two polysaccharides, 20–30% amylose and 70–80% amylopectin, both consisting of long glucose chains connected by α(1–4) glycosidic bonds.^5^ Amylose is linear, with only seldom branches, while amylopectin has high level of branching through α(1–6) glycosidic bonds.^5^ Although amylose is able to form both A- and B-type crystalline polymorphs, in starch granules, it folds up in helices with amorphous characteristics, while the amylopectin forms double-helices that constitute the crystalline structure of native starch.^5^ This very complex starch structure does not work well for pharmaceutical formulations; hence, extensive processing methods have been developed to make more well-defined starch particles.^6^

Already, many uses of modified starch as pharmaceutical agents exist on the market or have shown promising research results; this includes starch as a filler in drug tablets,^7^ starch particles for transdermal drug delivery,^8^ controlled release of small organic drugs,^9^ embolization,^10^ encapsulation of probiotics,^11^ and many other purposes.^12^ Carbohydrates furthermore stabilize proteins in dry states, enabling freeze-drying, which vastly prolongs the shelf life of drugs.^13^

A modified starch compound that has been hypothesized to be useful as a protein carrier is the degradable starch microsphere (DSM) from Magle-Chemoswed described in refs (14) and (15). DSMs are produced from the acid hydrolysis of potato starch followed by cross-linking. This process creates spherical starch particles of 25 μm to 1 mm in diameter when hydrated. The structural changes upon hydration of DSM have previously been studied by combination of small-angle X-ray scattering (SAXS) and other techniques.^15^ In the dry state, Digaitis et al.^15^ revealed DSM cores that were porous and shells that were dense but still contained wide pores, except for the very outer crust, which was nonporous. In the wet states (>17.5% H_2_O content), Digaitis et al.^15^ described the porosity with a static correlation length (SCL) and a dynamic correlation length (DCL), related to distances between cross-links and starch chains, respectively. Both correlation lengths increased with an increasing water content.

If DSMs are to become feasible protein carriers that can be used for controlled release, proteins must be able to enter the DSMs and remain stable and natively folded inside the hydrated particles. In this study, we used lysozyme as a model protein to test whether it gets absorbed by DSMs. Lysozyme is one of the most widely investigated proteins; hence, many articles have been published on its structure and properties in a wide range of states.^16−21^ Of particular relevance for the current study are small-angle scattering studies over large concentration ranges and under varied conditions.^17,18,22^ While lysozyme interacts with and degrades peptidoglycans^23,24^ and chitin,^25^ the interactions strongly depend on the *N-*acetyl group of the substrates,^26^ which is not present in starch or disaccharides such as sucrose and trehalose. It has been shown that the presence of sucrose, promotes stabilization of lysozyme conserving its native state in a solid sucrose matrix.^27^ Although studies of lysozyme interactions with modified starch materials were reported in the literature before,^28−30^ an experimental evidence of preservation of native structure of lysozyme in starch-based materials has not yet been presented.

To investigate how much protein is absorbed and released by DSMs, we did an absorption–desorption experiment, measuring the lysozyme content in the phases with UV–vis. The structural interactions between hydrated amorphous starch microspheres and protein were investigated with SAXS combining an ellipsoidal model to describe the shape of lysozyme with a Gauss-Lorenz gel model^31^ for the starch chains.

## 2 Materials and Methods

### 2.1 Materials

Two different kinds of dry DSM particles were provided by Magle-Chemoswed (Malmö, Sweden); the two DSM batches have respective average diameters of 400 and 580 μm in the swollen state. They were produced by water in oil emulsion cross-linking acid-hydrolyzed potato starch with epichlorohydrin. Prior to use, the DSM was dried at room temperature in an Abderhalden’s drying pistol with molecular sieves for 48 h. The properties of the DSM particles were described in detail in Digaitis et al.^15^ Lysozymes from chicken egg white (Lot L6876) and potassium phosphate monobasic were bought from Sigma-Aldrich. Potassium phosphate dibasic was purchased from Merck. All experiments used water of the Milli-Q quality.

### 2.2 Sorption and Desorption

#### 2.2.1 Swelling in Water

The swelling ratio of the DSM particles was estimated by measuring the mass of dry starch samples; the particles were then fully hydrated by addition of excess water. After filtration with Macherey Nagel filter paper (Düren, Germany. Catalogue no. 531018), the particles were measured gravimetrically again, and the swelling ratio was calculated.

#### 2.2.2 Sorption of Protein, UV–vis

The amounts of lysozyme absorbing to and released from DSMs were investigated by incubation of 2 wt % DSM particles with different concentrations of lysozyme. Figure S1 schematically shows the procedure. The samples were mixed on a rotator (Tube Revolver Rotator, ThermoFisher Scientific) for at least 4 h before centrifugation at 4000 rcf (using a Heraeus, Multifuge 3 S-R with a Swing-out rotor (75006445)), which sediments the DSM. The amount of lysozyme absorbed was calculated by measuring the concentration of lysozyme in the supernatant with UV absorbance at 280 nm (on a Shimadzu UV-1800 UV–vis Spectrophotometer). In advance, a standard curve for lysozyme absorbance at this wavelength was prepared by measuring lysozyme at different concentrations in H_2_O.

The total mass of lysozyme in the sample, *m*_Lys_^(total)^, is a sum of the lysozyme in the liquid phase and in the DSM phase. This gives the equation:
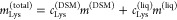
1where *c*_Lys_^(DSM)^ and *c*_Lys_^(liq)^ are the concentrations (weight fractions) of lysozyme in the DSM and liquid phases, respectively, and *m*^(DSM)^ and *m*^(liq)^ are the masses of the respective phases.

The concentration of lysozyme in the hydrated starch particles is hence given by
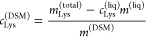
2

*m*^(DSM)^ is calculated from the dry mass of DSM at the start of the experiment multiplied by the swelling factor of 5.0. The mass of liquid *m*^(liq)^ is then calculated by subtracting *m*^(DSM)^ from the total mass of the two-phase system.

The concentration of lysozyme per dry mass of starch, *c*_Lys_^(DSM dry)^ is calculated as follows:
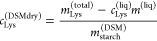
3

### 2.3 Small-Angle Scattering

**Figure 1:**
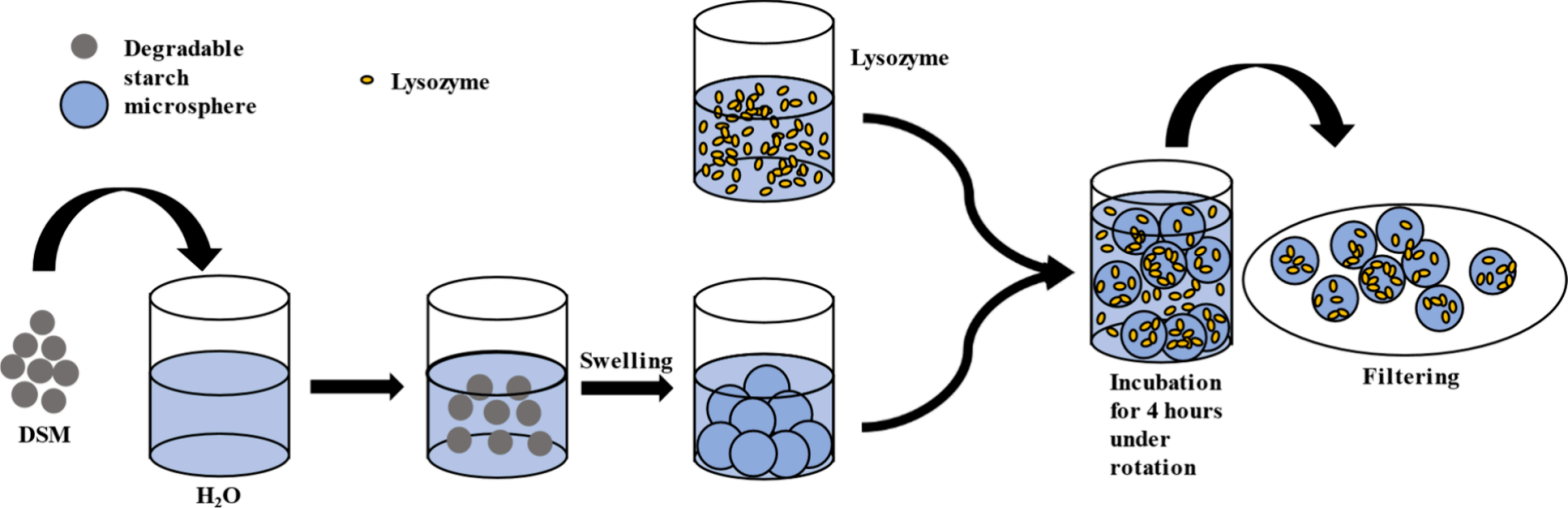
Preparation of the lysozyme–DSM samples for SAXS. Dry DSM particles are suspended in pure water. After swelling, dissolved lysozyme is added, and the mixture is incubated under rotation. The DSM particles with absorbed lysozyme are isolated by filtering the mixture, removing external liquid. The particles were subsequently transferred to a glass capillary for SAXS.

A schematic explanation of the preparation of DSM–lysozyme samples for SAXS experiments is shown in Figure 1. In this protocol, 4 wt % dispersions of DSM in H_2_O were prepared before addition of an equal mass of H_2_O (for pure DSM samples) or lysozyme solutions to different final concentrations (1–10 wt %, 10–111 mg/mL), giving a final DSM concentration of 2 wt %. The mixtures were incubated on a rotator (Tube Revolver Rotator, ThermoFisher Scientific) for at least 4 h, before they were filtered with folded filter papers, from Macherey Nagel (Düren, Germany. Catalogue nr. 531018). The filtered particles were transferred to 1.0–1.5 mm thick glass capillaries from Hilgenberg (Malsfeld, Germany. Catalogue nr. 4007615) with a sterile needle. Lysozyme (1–10 wt %) in H_2_O and 0.5–4 wt % lysozyme in 50 mM potassium phosphate buffer pH 7.4 were likewise prepared.

Small-angle X-ray scattering measurements were done on a Xenocs Xeuss 3.0 instrument (Xenocs, Grenoble, France) with a GeniX3D Cu Kα source (wavelength λ = 1.54189 nm), two scatterless slits for beam collimation, and a Pilatus 300 K detector (Dectris, Switzerland). Intensities, *I*(*q*), were recorded as a function of the scattering vector *q*, defined as *q* = 4πsin θ/λ, where 2θ is the scattering angle and λ is the wavelength. Data were collected at two sample-to-detector distances, 290 and 1800 mm, with exposure times of 3600 s at each distance, yielding useable data in the *q*-range ≈0.004 to ≈1.3 Å^–1^. Samples were measured at 25 °C. The scattering of empty capillary and water was used as a background. The data processing was done with the XSACT 2.4 software (Xenocs, Grenoble, France) as well as home written scripts in MATLAB (MathWorks, Natick, Massachusetts, USA).

### 2.4 SAXS Data Modeling

All modeling was done with SasView software (version 5.0.5)^32^ using build-in models and combinations of build-in models.

To determine the lysozyme form factor, a SAXS data set of lysozyme at infinite dilution in 50 mM potassium phosphate buffer pH 7.4 was generated using the experimental data from four different concentrations of lysozyme (0.5–4 wt %) in 50 mM potassium phosphate buffer pH 7.4; the program ALMERGE^33^ from the ATSAS 3.0 software package^34^ was used to generate this extrapolated data set. The form factor was then fitted with an ellipsoidal model. For 1D SAXS data, the scattering amplitudes *A*(*q*) from the form factor *P*(*q*) of an ellipsoid^35^ are given by
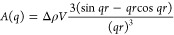
4with
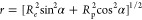
5where *R*_e_ and *R*_p_ are the equatorial and polar radius of a prolate ellipsoid, respectively. The volume *V* of the ellipsoid is given by
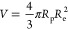
6

The SAXS data set of lysozyme at infinite dilution was also fitted with the calculated scattering of the crystal structure (1DPX^36^) using the software CRYSOL 3.0^37^ from the ATSAS 3.0 package.^34^

As lysozyme has a net charge of +8 at pH 7, SAXS data at higher concentrations of lysozyme in H_2_O are affected by the repulsion between the molecules. These data were therefore fitted with a *P*(*q*) × *S*(*q*) model where *P*(*q*) is the form factor as determined for the infinite dilute samples (i.e., all form factor parameters were fixed when modeling higher lysozyme concentrations), and *S*(*q*) is the Rescaled Mean Spherical Approximation (RMSA) structure factor for charged spheres as described by Hayter and Penfold.^38^

The scattering from DSM molecules was modeled with the same Gauss-Lorenz gel model^31^ approach as in Digaitis et al.,^15^ except we included the scattering contribution from the solid–liquid interface as a power law within the model. The full model is therefore,
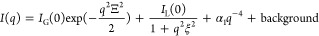
7where Ξ is the SCL, related to cross-links, ξ is the DCL, related to distances between fluctuating starch polymer chains, *I*_G_(0) and *I*_L_(0) are the respective scaling factor for the two correlation lengths, and α_1_ is the scaling factor for the interface scattering contribution.

For the mixtures of DSM and lysozyme, the respective models were combined; however for the lysozyme contribution, only the vol % for the structure factor and an overall scaling factor (α_2_) were fitted, and all other lysozyme form factor and structure factor parameters were fixed to what were obtained from the pure lysozyme data sets. The combined model is,
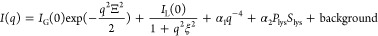
8

### 2.5 Optical Microscopy

Images of DSM samples were taken through a Nikon Optiphot (Tokyo, Japan) optical microscope equipped with a DS-U1 digital camera. The hydrated DSM samples were placed on optical glass slides to observe the overall shape and dimensions of the particles. Also, images were taken directly on SAXS glass capillaries containing the DSM samples to see the effect of a crammed environment on the shape.

## 3 Results

### 3.1 Lysozyme Absorption by DSM

To accurately measure the amount of lysozyme that DSM particles can take up, the mass of hydrated DSM needed to be known, we hence gravimetrically determined that the DSM mass increased 5-fold (5.0 ± 0.7) upon hydration. The obtained gravimetric swelling ratio *m*^DSM^/*m*_starch_^(DSM)^ is higher than the value recalculated from the volumetric swelling ratio reported by Digaitis et al.,^15^ probably due to porosity of DSM in the dry state. Indeed, the gravimetric swelling ratio *r*^*m*^ is related to the volumetric swelling ratio *r*^*V*^ as follows:
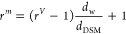
9

Hence, one might expect that the gravimetric ratio can be lower, because the density of starch is higher than the density of water. However, for these calculations, the “geometrical” density is relevant, which for DSM is about 0.88 g/cm^3^ due to the dry porosity of the amorphous starch microspheres.^15^

**Figure 2:**
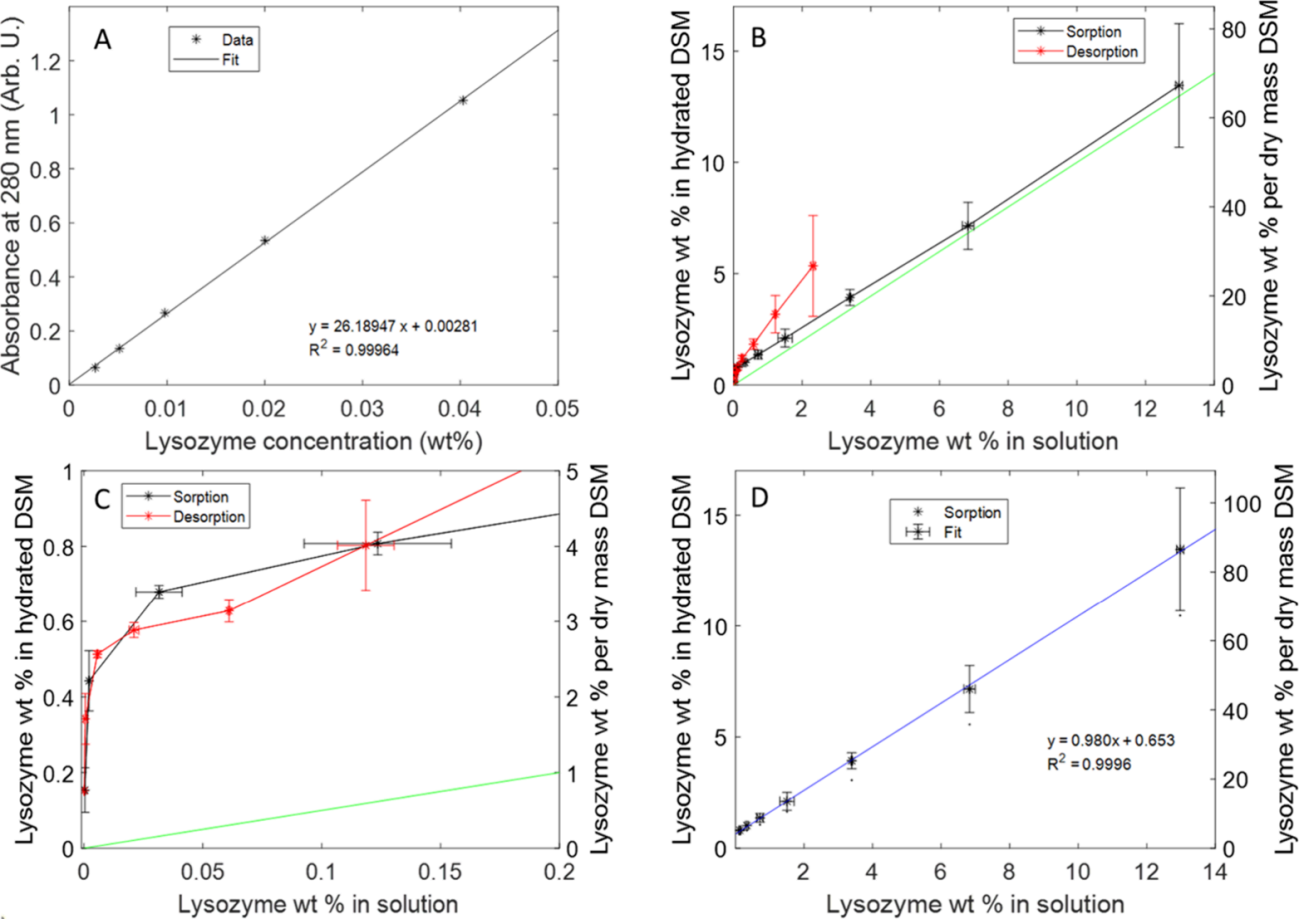
Sorption–desorption of lysozyme to DSM. (A) Dilute lysozyme solutions were measured at 280 nm on a UV–vis instrument (*), fitted with a linear model (−). The fitting parameters are used to calculate lysozyme concentrations in the DSM–lysozyme samples. (B–C) The calculated wt % of lysozyme in hydrated DSM vs the concentration in the solution (outside DSMs) after sorption (black) or after desorption (red). The right *y*-axis shows the concentration of lysozyme per dry DSM mass. Panel B contains all data, while panel C zooms in on the lowest lysozyme concentrations. The green line visualizes how the expected data would look if there was an equal amount of lysozyme within DSM and in the solution. Especially at lower concentrations of lysozyme, there is significantly more protein within the DSM compared to in the solution. Except at low concentrations (<0.5 wt % lysozyme in DSM), the data for both sorption and desorption approximate a linear relation. Panel D shows the linear fit to the sorption data, ignoring the 3 lowest concentrations. The fitting parameters are used for determining Lysozyme concentrations in the SAXS data analysis and are hence for lysozyme wt % in hydrated DSM versus in solution.

To test if lysozyme is absorbed and released by DSM particles, a sorption–desorption experiment was performed by mixing DSM with different concentrations of lysozyme in water. After sedimentation of the DSM particles, the supernatant was measured with UV–vis at 280 nm (tryptophan absorption), and from the readings, the amount of lysozyme in the DSM vs the supernatant was calculated using eq 2, based on a standard curve from measurements of lysozyme alone (Figure 2A). The particles were then resuspended in water, and the supernatant was measured by UV spectroscopy to see the amount of desorbed lysozyme. The sorption–desorption results are presented in Figure 2B–D, while the sample experimental procedure is sketched in Figure S1.

Interestingly, we saw that, at low concentrations of lysozyme (Figure 2C), all of the protein enters or binds to the DSM, with minimal protein left in solution. At higher concentrations of lysozyme, however, the correlation approaches a linear relationship between lysozyme in solution and within the DSM (Figure 2D). This indicates that, at lower lysozyme contents, the absorption is driven by stronger protein–starch interactions. These may be hydrophobic interactions and also involve electrostatic contribution if a small amount of negatively charged groups is present in DSM. At higher lysozyme concentrations, further accumulation of lysozyme in DSMs is counteracted by protein–protein electrostatic repulsion. As a result, at high protein contents, lysozyme concentrations in DSM and in the surrounding liquid are relatively similar.

### 3.2 Scattering of Free Lysozyme in Water

To get a structural understanding on the absorption of lysozyme into DSM and the potential interactions between the protein and starch, we investigated the individual components and the protein–DSM complex using SAXS. First, we characterized the form factor of lysozyme by SAXS measurements at low concentration in 50 mM potassium phosphate, pH 7.4. The data can be fitted with calculated scattering of the crystal structure of lysozyme (1DPX^36^), but to get simple tunable parameters, we also fitted the data with an ellipsoid model (both fits shown in Figure S2, parameters given in Table S1), yielding a good fit (χ^2^ = 3.57) with polar and equatorial radii, 27.25 and 15.06 Å, respectively, that both agree with those found in the literature.^17^ When excess liquid is removed from the space between hydrated DSMs, the weight percentage of starch is 20 (calculated from gravimetric measurements); this necessitates high concentrations of lysozyme in the DSM–lysozyme experiments to get visible scattering from the protein. We therefore also measured lysozyme samples at high concentrations with SAXS, to see the structure factor of concentrated lysozyme in water. These experiments, as well as subsequent experiments with DSMs, were done in pure water rather than in buffers with salt. This was in part to ensure high solubility of lysozyme and in part to make the data comparable to previous studies on both DSM^15^ and lysozyme^17,18^ under similar conditions. The SAXS data from the highly concentrated solution of lysozyme were then fitted with the ellipsoidal model as a form factor, fixing the parameters yielded from the low-concentration experiment, combined with the RMSA structure factor model for charged spheres.^38^ We found that this approach gave good fits (Figure S3A, Table S1); however, the modeled volume percentages (vol %) were always higher than the actual vol %, especially at 1–5 wt %, where the modeled vol % was around twice as high as the actual (Figure S3B). This shows that the model does not perfectly describe the lysozyme structure factor, possibly because RMSA artificially rescales the volume fraction as pointed out by Pandey and Tripathi,^39^ or because the RMSA is for spherical solid particles with evenly distributed charges, while lysozyme has an ellipsoidal shape with disproportionally many solvent-facing, positively charged arginine residues. However, as the model still fitted the data very well, we found it satisfying for investigating relative changes in apparent vol % upon absorption in DSMs.

### 3.3 Scattering of Hydrated DSM Particles

Prior to the SAXS studies of DSM, with or without absorbed lysozyme, an external liquid was removed by filtration. This was done to ensure that the contribution of lysozyme to the scattering would come only from lysozyme associated with the starch particles. The sample preparation of the lysozyme–DSM complex is summarized in Figure 1. The SAXS experiments with hydrated DSM without the presence of lysozyme were performed for comparison and proper subtraction of the contribution from the starch particles.

**Figure 3:**
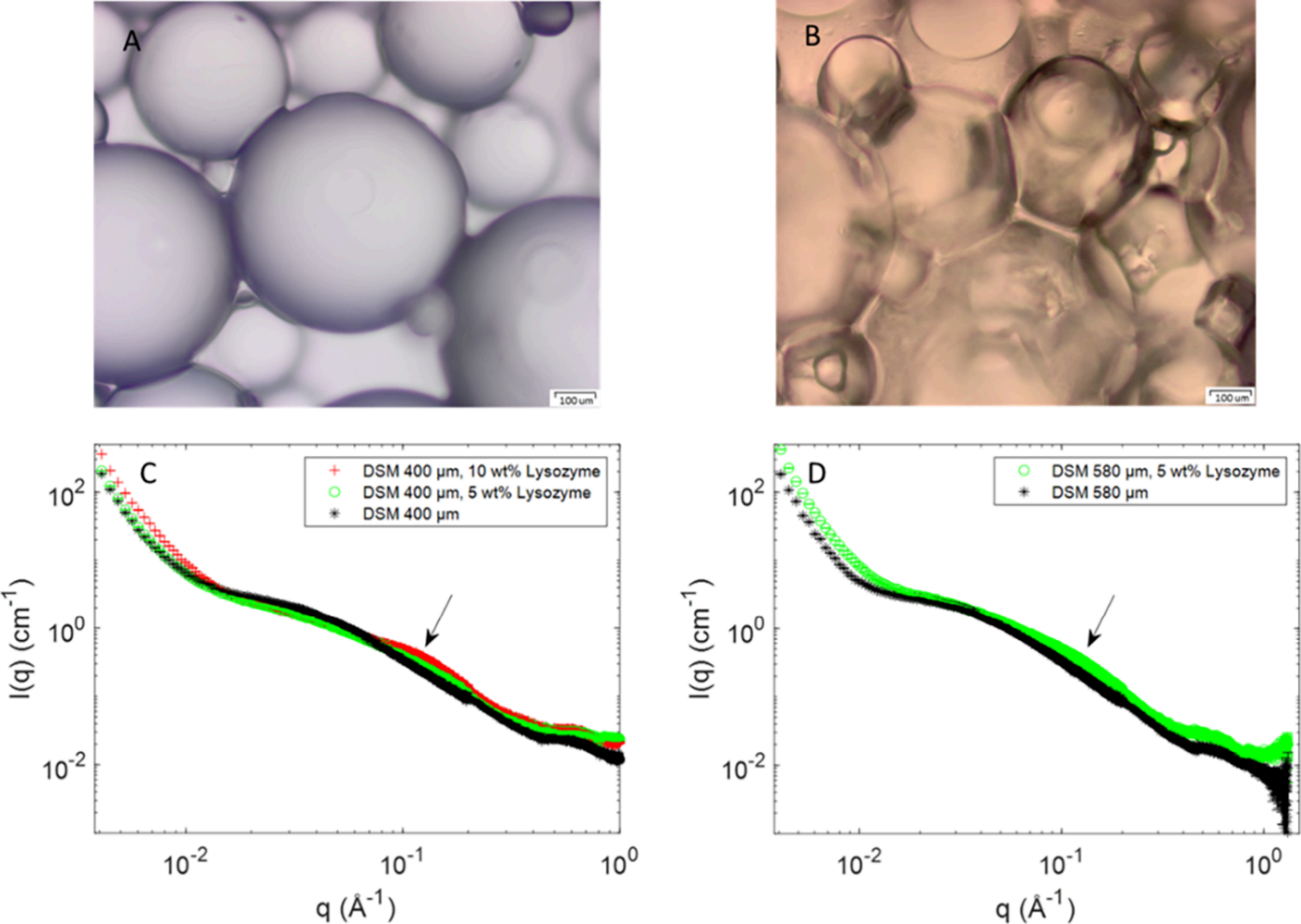
Scattering from DSM with and without lysozyme. (A, B) Microscope image of DSM particles (average swollen diameter: 400 μm) in the hydrated state used in the SAXS experiments, calculated to be 20 wt % DSM. Panel A shows the particles imaged on glass slides, showing a morphology consistent with what has previously been reported. Panel B shows the particles imaged in a SAXS glass capillary, where a crammed environment deforms the particles slightly. (C, D) SAXS data of DSM with or without absorbed lysozyme. In panel C, DSM particles of an average swollen diameter of 400 μm were used, while 580 μm was the average diameter of the particles used in panel D. Arrows in panels C and D point to the bump that appears in the presence of lysozyme, coming from the form factor of the protein.

Through weight measurements, the DSM content in the filtered samples was determined to be 20 wt %. Microscope pictures (Figure 3A) also looked similar to the those obtained by Digaitis et al.^15^ at the same system composition. At this concentration, the spheres are deformed in the cramped environment of the SAXS capillary, eliminating possible empty space between particles, as seen in microscope images of the particles within the capillary (Figure 3B).

For modeling the SAXS data of DSM, we used the same Gauss-Lorentz gel model as used by Digaitis et al.,^15^ except we also modeled the scattering contribution from the interfaces between starch and water using a power law. This allowed fitting data for a broad *q*-range (0.005–0.45 Å^–1^). For the pure hydrated DSM samples, we obtained DCL values of 31.38 and 32.70 Å for the DSM samples with 400 and 580 μm as average diameters, respectively (Table S2). This is similar to what Digaitis et al.^15^ got at the same concentration (around 30 Å). The obtained values of the static correlation length (SCL) are 520.9 and 365.4 Å for the smaller and larger DSM particles, respectively (see Table S2), which is higher than those reported by Digaitis et al.^15^ The reason for this is probably in the difference in the models at low *q* values. While Digaitis et al. did not model the scattering from interfaces (as the scattering at the lowest *q*-values were unimportant for their analysis), we explicitly included it in our model. Since the interface scattering contributes to the scattering intensity in the same *q* range as scattering from distances between cross-links, its addition in the model affects the results.

### 3.4 Structural Investigation of the DSM–Lysozyme Complex

When lysozyme is present in the DSM, an extra bump on the SAXS curve is present at *q* = 0.10–0.12 Å^–1^ (Figure 3C,D), where it is also observed for pure lysozyme (Figure S3). As the scattering clearly shows contributions from both the DSM and lysozyme, we combined the models from the DSM and lysozyme analyses, giving the full model as shown in eq 8.

We hypothesize that the lysozyme can be unevenly distributed within the DSM, because of the differences in density and porosity between the core and shell of DSMs as previously described for the dry state,^15^ and because of the internal structure of hydrated DSM, where some parts might be less dense than others. Moreover, as we have shown above, the lysozyme volume fraction obtained in fitting using the RMSA structure factor can deviate from the actual protein concentration. We therefore used the lysozyme volume fraction in the RMSA structure factor as a fitting parameter in the modeling, while all other parameters for the form factor and structure factor of lysozyme were fixed to those obtained in the modeling of the pure lysozyme samples at corresponding concentrations, except a scaling factor α_2_ for the overall contribution of the lysozyme scattering. For modeling starch chains, the parameters for the SCL and DCL were also fitted, as these can be affected by the presence of lysozyme. Likewise, the scaling factor α_1_ for the power law describing the scattering from interfaces needed to be fitted, as this contribution varied significantly between samples, probably depending on where the X-ray beam hit the DSM particles.

**Figure 4:**
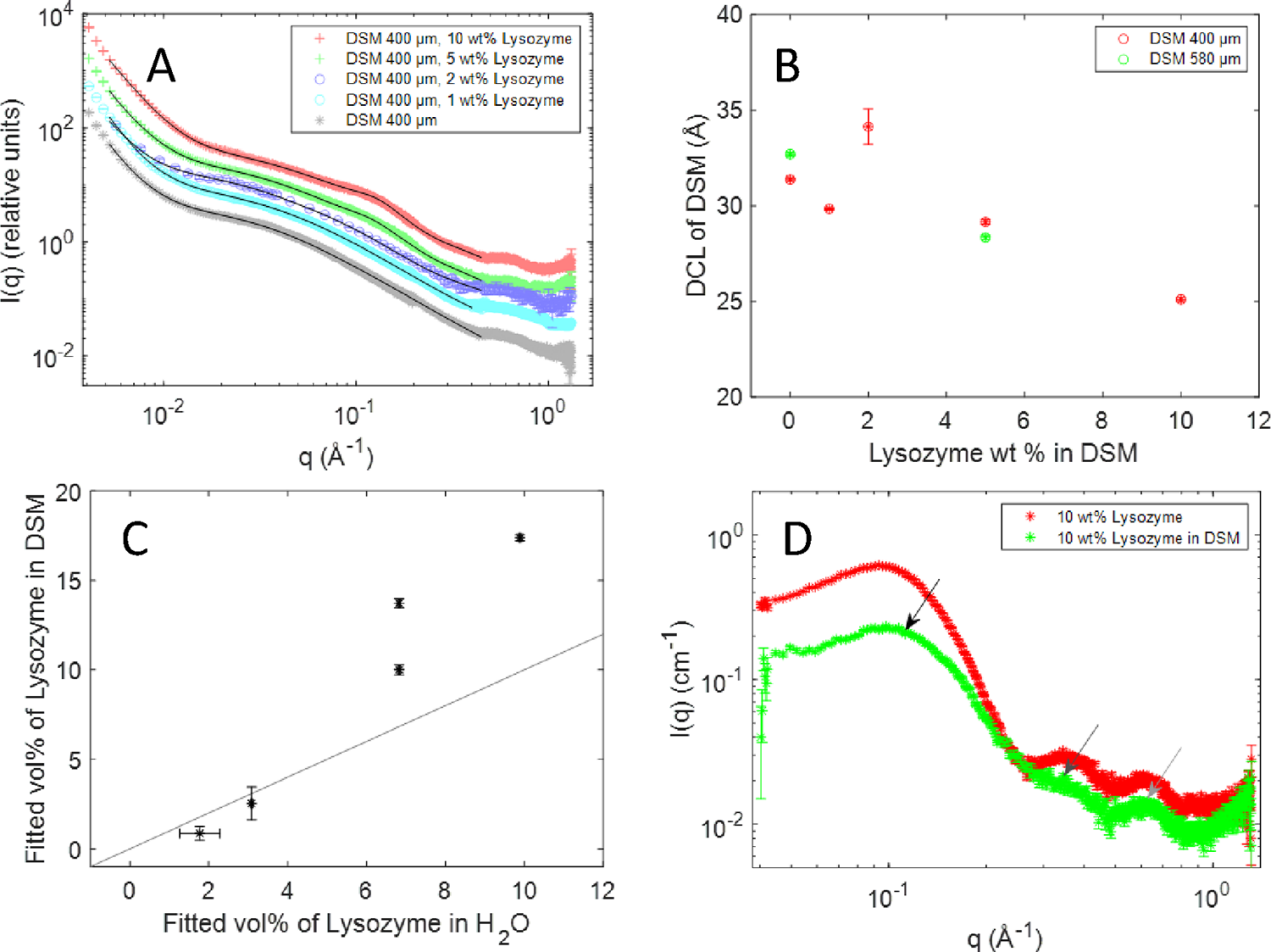
Analysis of the scattering from DSM–lysozyme. (A) SAXS data of DSM with (or without) lysozyme at different concentrations fitted with the lysozyme–DSM model (black lines). Fitted parameters are summarized in Table S2. (B) DCL as a function of the lysozyme concentration in DSM. (C) Fitted vol % of lysozyme in DSM vs fitted vol % of lysozyme in H_2_O. The gray line shows an expected trend if fitted parameters were identical in DSM and in water. (D) SAXS data of 10 wt % lysozyme in water (red) and scattering of 10 wt % lysozyme in DSM, where the experimental DSM scattering is subtracted (green). Arrows point to characteristic lysozyme peaks. From left to right: black arrow: protein–protein interaction. Dark gray arrow: the first submaximum of the form factor. Light gray arrow: interactions between α-helices.

The model fitted the SAXS data very well in the *q*-range up to 0.4–0.5 Å^–1^ (Figure 4A, Figure S4). The scattering intensity at higher *q* values is affected by scattering from individual glucose rings of starch^40^ and also the internal structure of lysozyme,^17^ which was not considered in the model. While the obtained values for the SCL were uncertain for the reasons described above, they all remained within the range 300–650 Å (See Table S2), being either similar or slightly larger than previously reported.^15^ Changes to DCL were subtle at lower lysozyme concentrations but dropped noticeably at higher lysozyme concentrations (Figure 4B). The observed correlation length change could potentially be explained by the effect of the ionic strength and pH that addition of polymer may cause. However, a recent study showed that the DSM density and degree of swelling undergo only minor changes upon variation of the aqueous solution properties.^41^ Hence, the observed DCL change can be explained by the lysozyme forcing the chains closer together to make space for the protein at higher concentrations. Considering that the DSM particles are ≈400 μm in diameter, there could be changes in the particles on distances too large for SAXS; however, the particles look very similar in the microscope whether in the presence or absence of lysozyme (Figure S5).

At lower concentrations of lysozyme, the modeled vol % of the protein in DSM is slightly lower than those obtained from modeling the protein alone at equivalent concentrations (Figure 4C), although from the sorption isotherm, it is expected to be 0.6 wt % higher (Figure 2D). Apart from possible effects of starch on protein–protein interactions, this small discrepancy may arise from the loss of lysozyme during the filtration step, which influences low protein concentrations more.

In contrast, at higher lysozyme concentrations, the modeled protein volume fraction in DSM is higher than in pure protein samples. This is in qualitative agreement with the sorption isotherm, which shows higher concentration of lysozyme in starch compared to the surrounding liquid (Figure 2). While starch is formally neutral, some charges can be introduced during the acid hydrolysis^15^ or cross-linkage. These charges (most probably negative) may play a role in electrostatic interactions with positively charged lysozyme, causing a constant shift of the nearly linear sorption isotherm by 0.6 wt %. Moreover, the lysozyme concentration increase in DSM compared to the surrounding liquid is stronger when seen from SAXS modeling than that from the sorption isotherm. This can be attributed to the uneven distribution of lysozyme inside the DSM particles. First, variation of lysozyme concentration may follow variation of density of the starch material. Second, lysozyme molecules may exhibit a local increase of concentration, in agreement with the above-observed fact that DCL of starch chains decreases with the increase of lysozyme concentration. In other words, our data suggest that the lysozyme-cross-linked starch system, being macroscopically homogeneous, may microscopically form starch-enriched and lysozyme-enriched regions.

### 3.5 State and Structure of Lysozyme within DSM

As we only saw limited changes in the starch parameters, we decided to also try subtracting the experimental DSM scattering from the mixture at mid-to-high *q*. This would reveal whether the lysozyme-characteristic SAXS peaks are preserved in the DSM-absorbed state.

We see in Figure 4D that the peak at ≈0.35 Å^–1^, corresponding to the secondary maximum of the lysozyme form factor,^17^ is still present, although less defined, possibly because of imperfect subtraction due to differences between DCL of starch in the presence and absence of lysozyme, the peak at ≈0.6 Å^–1^ corresponding to distances between alpha helices is also preserved. This confirms that the protein is in its native state. The maximum of the protein–protein interaction peak is slightly shifted in the DSM-absorbed state, from a maximum at *q* = 0.095 to 0.103 Å^–1^, this is consistent with an increase in the vol % of the protein, the peak also appears less sharp, which can be attributed to the inhomogeneous distribution of lysozyme within DSM.

As our model fits the lysozyme–DSM data very well without the need for extra lysozyme–DSM cross-terms, and since we only see limited changes to the fitting parameters of both DSM and lysozyme, the starch–lysozyme interactions in the hydrated system are weak, perhaps with the exception of a small fraction of lysozyme molecules, which results in a constant shift of the sorption isotherm. The slight preferential absorption may be due to either trace negative charges on the DSM arising during the production of the particles or minor hydrophobic interactions between the protein and starch. Moreover, the model fitting indicates that protein molecules exhibit a trend to avoid starch chains and accumulate close to other protein molecules. This correlates with the idea of preferential hydration, i.e., preferential exclusion^42−44^ of cosolvent (starch in this case) from the surface of protein. The effect, however, is not very strong since it does not prevent protein molecules from absorption in amorphous starch microparticles.

## 4 Conclusions

A compound can be a suitable protein carrier only if it can absorb proteins in sufficient concentrations. Furthermore, it is essential that the carrier does not drastically alter the conformation of the absorbed protein. By using lysozyme as a model protein, we have demonstrated that DSM can absorb proteins at concentrations exceeding the concentration in the surrounding liquid. With SAXS, we have shown that incorporation of lysozyme in the amorphous starch particles has only minor effects on the polysaccharide network, as manifested by a small reduction of the characteristic chain–chain distance. The lysozyme structure was successfully preserved within the DSM, as evidenced by the scattering pattern corresponding to the native form factor and the presence of peaks arising from secondary structure elements.

SAXS modeling data suggested slightly uneven distribution of lysozyme within the DSM, partly due to the density variation between the core and shell of DSM, but also due to spontaneous formation of protein- and carbohydrate-enriched domains. Overall, our results support the potential of DSMs as protein carriers. We showed that protein molecules can be incorporated in cross-linked starch microparticles without disturbing the protein’s native structure. Moreover, since the lysozyme absorption does not rely on electrostatic or specific interactions, the absorption will likely be possible for proteins having different charges and other properties; however, proteins that do exhibit specific interactions with starch will likely show a different absorption behavior. Finally, the ability of the starch chains to respond to the addition of the protein by varying interchain distance opens possibilities for absorption of proteins having sizes exceeding the unperturbed interchain distances.

## Supporting Information Available

Tables of all modeling parameters for the fits presented in the paper; graphs of SAXS data for lysozyme and DSM–lysozyme system (average diameter 580 μm), including model fits; graphic presentation of the sample preparation for the UV sorption–desorption experiment; and microscopy images of DSM and DSM–lysozyme system (PDF)

## Abbreviations

DSM: degradable starch microspheres
DCL: dynamic correlation length
RMSA: Rescaled Mean Spherical Approximation
SAXS: small-angle X-ray scattering
SCL: static correlation length
UV–vis: ultraviolet–visible spectroscopy
vol %: volume percentage
wt %: weight percentage
