# Supplementary material for: A Structural Study on Absorption of Lysozyme in Amorphous Starch Microspheres
Source: Mol Pharm. 2024 May 13;21(7):3416–24. doi: 10.1021/acs.molpharmaceut.4c00135 (PMC11220755; doi:10.1021/acs.molpharmaceut.4c00135)
Supplement: Supplementary file 1 — mp4c00135_si_001.pdf [file mp4c00135_si_001.pdf]

Supplementary information

A structural study on absorption of Lysozyme in amorphous starch microspheres

Henrik Vinther Sørensen[a,b], Nedim Krcic[c], Ian George[c], Vitaly Kocherbitov[a,b*]

[a]Department of Biomedical Science, Faculty of Health and Society, Malmö University, Malmö, 20506, Sweden

[b]Biofilms Research Center for Biointerfaces, Malmö University, Malmö, 20506, Sweden

[c]Magle Chemoswed AB, Agneslundsvägen 27, Malmö, 21215, Sweden

*Corresponding author: Vitaly Kocherbitov (vitaly.kocherbitov@mau.se)

Table S1: Obtained parameters from modelling Lysozyme data.

Sample	q-range (Å^{-1})	Polar radius (Å)	Equatorial radius (Å)	Vol%	Salt conc. (M)	χ^2
Lysozyme at infinite dilution	0.017-0.5	27.25 ± 0.28	15.06 ± 0.05	N/A	N/A	3.57
Lysozyme 1 wt %	0.023-0.5	27.25*	15.06*	1.77 ± 0.51	0.0056 ± 0.0098	0.33
Lysozyme 2 wt %	0.007-0.5	27.25*	15.06*	3.08 ± 0.03	0.0026 ± 0.0003	2.13
Lysozyme 5 wt %	0.013-0.5	27.25*	15.06*	6.83 ± 0.03	0.0063 ± 0.0003	3.73
Lysozyme 10 wt %	0.010-0.5	27.25*	15.06*	9.89 ± 0.04	0.0200 ± 0.0005	5.02

** Polar and equatorial radius was not a fitting parameter in higher concentrations of Lysozyme.*

Table S2: Parameters obtained from modeling SAXS data of DSM and DSM-Lysozyme mixtures.

Sample	q-range (Å^{-1})	Power law scaling factor	SLC scaling factor	SLC (Å)	DLC scaling factor	DLC (Å)	Lysozyme scaling factor	Vol% Lysozyme	χ^2
DSM (398 µm)	0.005-0.45	3.02×10^{-8} ± 0.02×10^{-8}	299.9 ± 68.8	520.9 ± 16.5	3.85 ± 0.01	31.38 ± 0.08	0.0	0.0	3.64
DSM (398 µm) + 1 wt% Lysozyme	0.005-0.40	4.31×10^{-8} ± 0.02×10^{-8}	515.1 ± 34.1	505.0 ± 5.0	4.20 ± 0.02	29.85 ± 0.07	0.0026 ± 0.0001	0.87 ± 0.38	5.13
DSM (398 µm) + 2 wt% Lysozyme	0.004-0.45	2.26×10^{-8} ± 0.2×10^{-8}	23.7 ± 35.5	439.0 ± 167. 0	4.58 ± 0.12	34.14 ± 0.93	0.0072 ± 0.0012	2.53 ± 0.91	1.87
DSM (398 µm) + 5 wt% Lysozyme	0.005-0.45	3.59×10^{-8} ± 0.02×10^{-8}	909.3 ± 38.7	614.7 ± 32.3	2.95 ± 0.01	29.16 ± 0.12	0.0120 ± 0.0002	13.72 ± 0.26	1.96
DSM (398 µm) + 10 wt% Lysozyme	0.005-0.45	6.80×10^{-8} ± 0.02×10^{-8}	915.7 ± 67.0	627.5 ± 55.4	2.44 ± 0.01	25.11 ± 0.11	0.0217 ± 0.0002	17.38 ± 0.17	2.05
DSM (578 µm)	0.005-0.45	9.25×10^{-9} ± 0.04×10^{-8}	182.2 ± 3.1	365.4 ± 1.7	4.00 ± 0.02	32.70 ± 0.09	0.0	0.0	5.33
DSM (578 µm) + 5 wt% Lysozyme	0.005-0.45	4.94×10^{-8} ± 0.03×10^{-8}	548.9 ± 34.3	467.8 ± 5.0	3.56 ± 0.02	28.34 ± 0.12	0.0119 ± 0.0002	10.00 ± 0.28	2.97

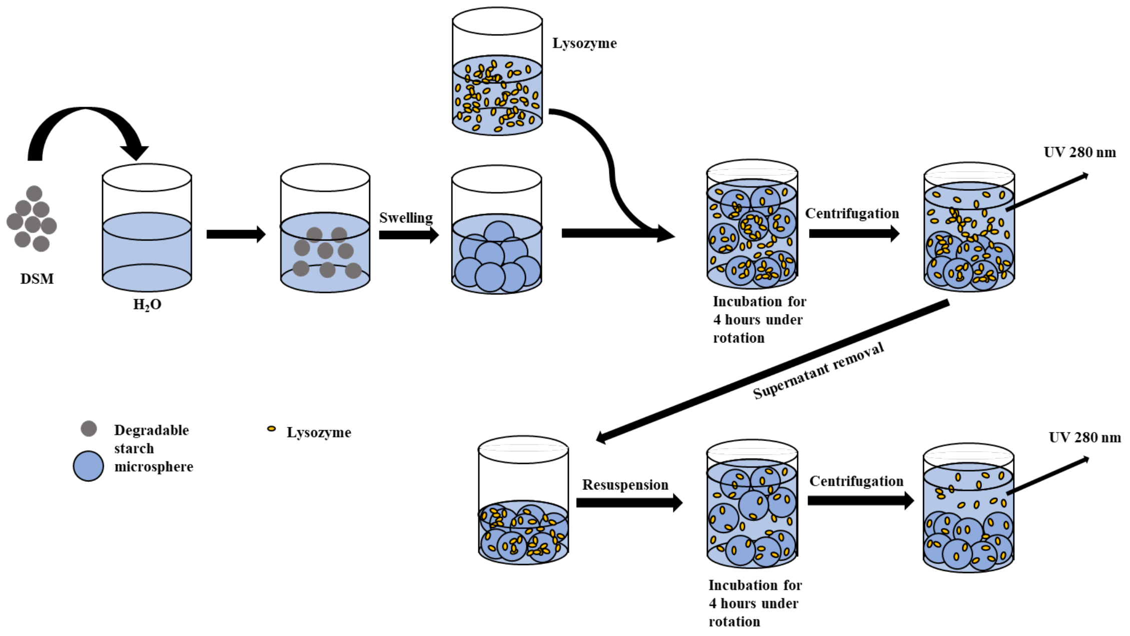

Figure S1: *Preparations of samples for UV measurements at 280 nm. Dry DSM was suspended in H_2O. After swelling, dissolved lysozyme was added to different concentrations. The mixture was incubated on a rotator for 4 hours. After centrifugation at 4,000 rcf, the supernatant was measured by UV-vis at 280 nm, to determine the lysozyme concentration in the supernatant, and thereby calculate the concentration in the DSM. The Supernatant was removed and the pellet was resuspended in H_2O, then mixed by rotation, and again centrifuged, before the supernatant was measured by UV-vis at 280 nm. Thereby enabling determination of the amount of released lysozyme and the amount still associated with DSM.*

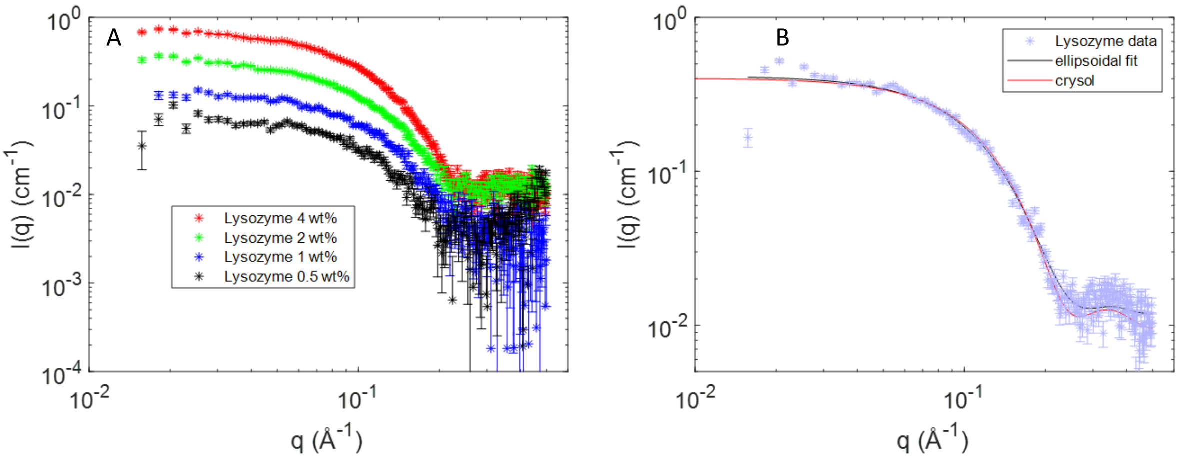

Figure S2: ***Lysozyme form factor analysis.*** *Lysozyme at different concentrations (**A**) were measured by SAXS, and the data was used to calculate a dataset at infinite dilution (**B**), which was fitted with crysol, using the crystal structure, and with an ellipsoid model. Parameters are summarized in Table S1.*

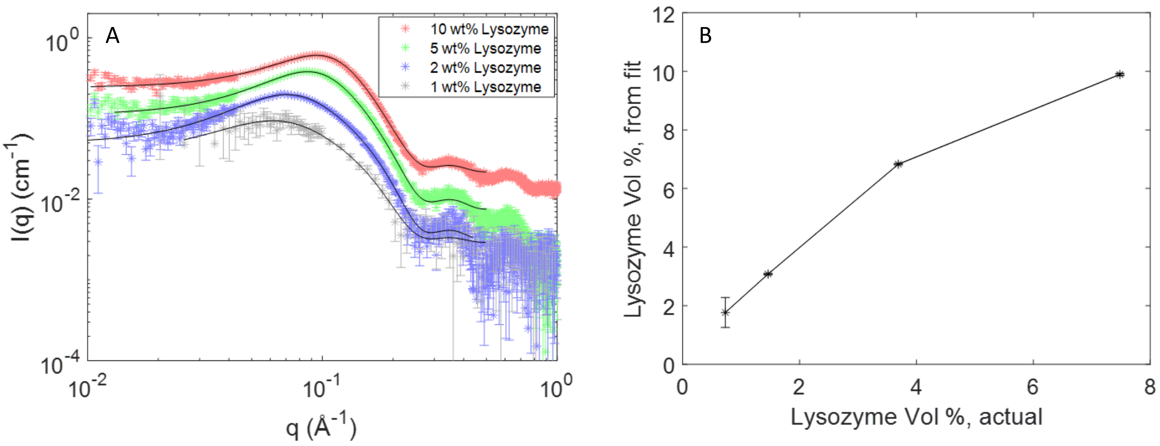

Figure S3: Scattering of lysozyme solutions at higher concentrations ***(A)*** *Higher concentrations of lysozyme were measured by SAXS to see the effect of structure factors on the lysozyme scattering. The data were fitted with an ellipsoid model multiplied by an RMSA structure factor (fits shown with black lines). Parameters are summarized in Table S1.* ***(B)*** *The fitted volume fractions from the RMSA structure factors vs. the actual volume fractions used in the experiment. The fitted values are generally higher than the actual.*

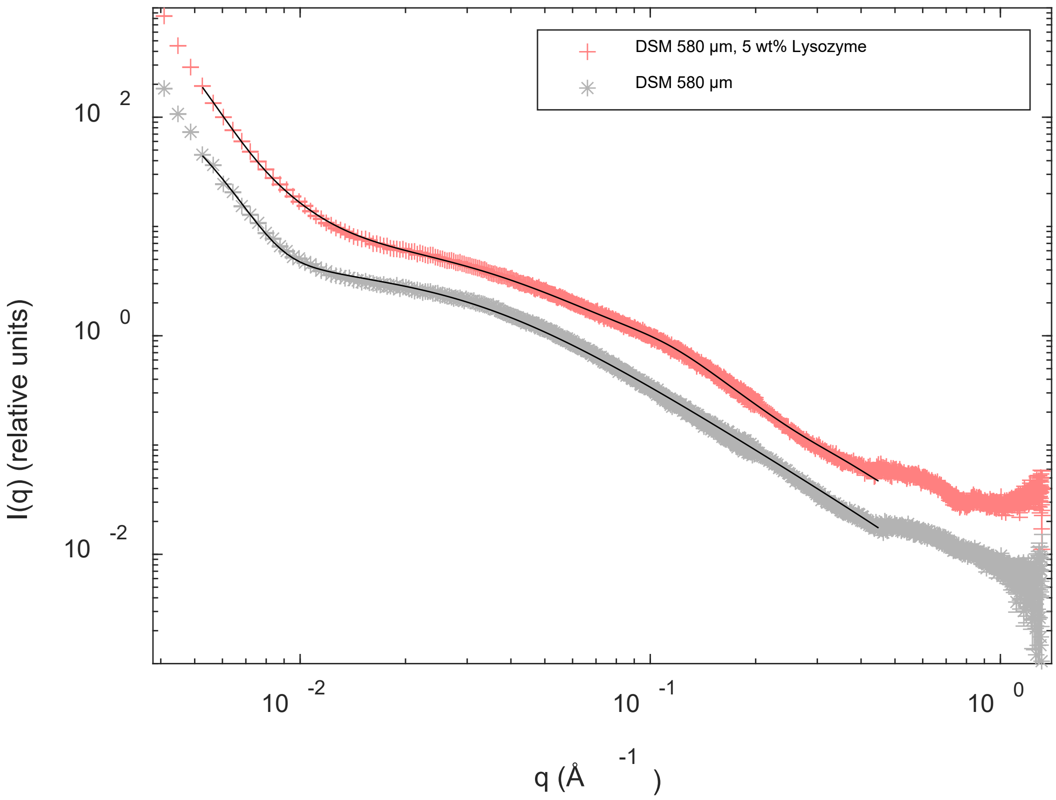

Figure S4: *SAXS data of DSM (average swollen diameter = 580 µm) with or without 5 wt% Lysozyme. The data were fitted with the Lysozyme-DSM model (black lines). Parameters are summarized in Table S2.*

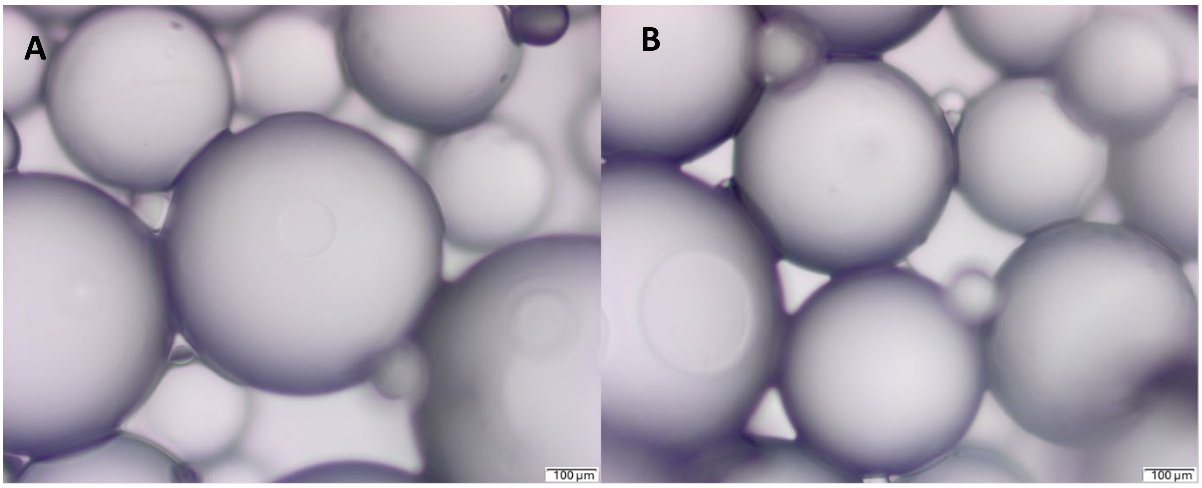

Figure S5: ***(A)*** *DSM 400 µm,* ***(B)*** *DSM 400 µm + 10 wt % lysozyme. No visible difference between DSM particles and DSM particles carrying lysozyme.*
